# Neutrocyte-to-lymphocyte ratio predicts the presence of a replicative hepatitis C virus strand after therapy with direct-acting antivirals

**DOI:** 10.1007/s10238-019-00561-y

**Published:** 2019-05-24

**Authors:** Anna Wróblewska, Beata Lorenc, Małgorzata Cheba, Krzysztof P. Bielawski, Katarzyna Sikorska

**Affiliations:** 1 Laboratory of Molecular Diagnostics, Intercollegiate Faculty of Biotechnology UG & MUG, Abrahama 58, 80-307 Gdańsk, Poland; 2 Pomeranian Center of Infectious Diseases and Tuberculosis, Smoluchowskiego 18, 80-214 Gdańsk, Poland; 3 Department of Tropical Medicine and Epidemiology, Department of and Tropical Medicine and Parasitology, Faculty of Health Sciences, Institute of Maritime and Tropical Medicine, Medical University of Gdansk, Powstania Styczniowego 9b, 81-519 Gdynia, Poland

**Keywords:** Occult hepatitis C infection, Replicative HCV-RNA strand, Interferon lambda, Direct-acting antivirals, Neutrocyte-to-lymphocyte ratio

## Abstract

Residual HCV-RNA can persist in liver tissue and peripheral blood mononuclear cells (PBMCs) long after antiviral therapy of chronic hepatitis C in patients repeatedly negative for viral RNA in serum. This occult infection associates with impaired immune response and the risk of lymphoproliferative disorders or progressive liver disease. There are currently no monitoring strategies for patients after treatment. We investigated if serum inflammation markers and interferon lambda (*IFNL*) genotype can be predictors of the presence of HCV-RNA and the replicative HCV-RNA (−) strand in patients who reached sustained virological response after interferon-free therapy. Forty-two consecutive patients who remained HCV-RNA negative in serum 24 weeks after the end of treatment (EOT) and during the follow-up were enrolled. Total HCV-RNA and HCV-RNA (−) strand were detected using ultrasensitive RT-PCR in PBMCs collected 12–15 months after EOT. Polymorphisms within *IFNL3*–*IFNL4* region (rs12979860 and ss469415590) were genotyped with allele-specific PCR. Viral RNA was found in PBMCs from 31 (74%) patients, and of those 29 (69%) were also positive for HCV-RNA (−). Neither normalization of alanine aminotransferase nor *IFNL* genotype predicted the presence of residual HCV-RNA. A significantly higher neutrocyte-to-lymphocyte ratio (NLR) 24 weeks after the start of treatment predicted elimination of replicative HCV-RNA strand (OR 0.23; 95% CI 0.10–0.86; *P* = 0.019). Patients with no HCV-RNA (−) in PBMCs showed a greater increase in neutrocyte count between EOT and baseline (*P* = 0.028). Lack of significant elevation of NLR after therapy with direct-acting antivirals could predict the presence of residual replicative HCV-RNA strand in PBMCs.

**Electronic supplementary material:**

The online version of this article (10.1007/s10238-019-00561-y) contains supplementary material, which is available to authorized users.

## Introduction

Secondary occult hepatitis C infection (OCI) is defined as the presence of HCV-RNA in liver tissue or peripheral blood mononuclear cells (PBMCs) in anti-HCV-positive patients repeatedly negative for serum HCV-RNA who reached a sustained virological response (SVR) after antiviral therapy [1]. Various types of lymphoid cells were shown to support HCV replication, being an important, long-term reservoir of the virus [1–3]. Viral infection of PBMCs in chronic hepatitis C (CHC) has a profound effect on immune cell function and carcinogenesis, leading to lymphocyte proliferative disorders, including mixed cryoglobulinemia and B cell non-Hodgkin lymphoma [4, 5]. Also persistence of residual HCV-RNA in PBMCs after therapy in SVR patients is linked with impaired immune responses [6, 7], as well as the risk of progressive liver disease and extrahepatic manifestations of HCV infection [1, 8].

The problem of OCI gained recently much attention with the introduction of interferon-free regiments for the treatment of CHC. Persistence of HCV-RNA in liver tissue after reaching SVR after direct-acting antivirals (DAAs) was shown in patients awaiting liver transplantation and in HCV-infected liver transplant recipients [9, 10]. The presence of HCV-RNA (−) strand, a signature of viral replication, was also documented in the latter group of patients [10].

The general prevalence of OCI after therapy with DAA and the mechanisms leading to viral persistence remain unknown. Viral replication and production of HCV particles occurs in OCI at a very low level, which makes persisting HCV-RNA undetectable with conventional clinical tests of serum samples. *IFNL* genotype is a known predictor of spontaneous or treatment-induced HCV clearance [11]. We aimed to investigate if basic immunological markers and *IFNL* genotype can be predictive of viral persistence after therapy with DAAs.

## Patients and methods

### Patients selection

Forty-two consecutive CHC patients infected with HCV genotype 1b, treated with DAA for 12 weeks, who completed therapy between March 2015 and August 2016, were enrolled. All patients reached SVR and remained HCV-RNA negative in serum 24 weeks after the end of DAA therapy (SVR24) and during the follow-up. Before treatment all individuals were DAA naïve, 10 were treatment naïve, and 32 previously underwent ineffective IFN-based therapy. Baseline characteristics of patients are shown in Table S1. Patients received either ombitasvir, paritaprevir, ritonavir, dasabuvir, ribavirin (OBV/PTV/r + DSV ± RBV), sofosbuvir, ledipasvir, ribavirin (SOF/LDV + RBV), or sofosbuvir with ribavirin (SOF + RBV). Blood morphology and biochemical analyses in blood were routinely performed at the beginning of therapy, after 4, 8, 12, 24 and 48 weeks as well as at the follow-up (after 60–72 weeks). For each of the time points mean platelet volume (MPV) was recorded and ratio of neutrocyte (neutrophil) to lymphocyte counts (NLR) as well as platelet-to-lymphocyte count ratio (PLR) were calculated for every patient. Systemic immune-inflammation index (SII) was computed as platelet count × neutrocyte count/lymphocyte count. Normalization of alanine aminotransferase (ALT) at the follow-up was defined as ALT < 40 IU/L. Detection of HCV-RNA was performed 12, 24, 48 weeks after therapy and at the follow-up with Cobas Amplicor (Roche) with detection limit 15 IU/mL.

The study protocol was approved by the Local Independent Bioethics Committee at the Medical University of Gdansk (NKEB 246/2011) in compliance with the Declaration of Helsinki. All participants provided written informed consent.

### Detection of occult HCV infection

Whole-blood samples were collected at the follow-up 12–15 months after the end of therapy (EOT). PBMCs were isolated using density gradient centrifugation and incubated for 72 h in the presence of mitogens: phytohemagglutinin-M and concanavalin A. Cells were stored in − 80 °C prior to total RNA isolation. For total HCV-RNA detection RNA was reverse-transcribed with RevertAid Kit (Thermo Fisher, USA) using random hexamers as primers. For detection of HCV-RNA-negative strand RNA was transcribed with Tth polymerase (Promega, Germany) and forward Tth_F primer. Tth polymerase possesses intrinsic Mn-dependent reverse transcriptase activity and stability in high temperatures, which enables highly specific synthesis of cDNA and helps to overcome problems with RNA secondary structures. Final HCV-RNA detection of both total and negative strand of HCV-RNA was performed with real-time PCR using LightCycler 480 SYBR Green I Master and LightCycler 480 (Roche Applied Science, Germany) with 5′UTR_F and 5′UTR_R primers. Detection limit of the final amplification step was ≤ 1.5 IU/µg RNA (≤ 5 viral genomic equivalents/µg RNA) as determined using dilutions of pSGR-JFH1 plasmid [12], containing whole HCV genome as a standard. During all the procedures both positive and negative controls were processed in parallel with patients’ samples. Specificity of PCR products was confirmed by melting curve analysis and sequencing.

Primer sequences and details of the HCV-RNA detection procedures as well as exemplary results of HCV-RNA detection are shown in Supplementary Material (pp. 3–4; Fig. S1).

### Genotyping

Genotyping of *IFNL3* rs12979860 and rs368234815 (*IFNL4* ss469415590) was performed as previously described [11].

### Statistical analysis

Statistical analysis was carried using data analysis software STATISTICA version 13.3 (StatSoft, Inc., USA). The software was also used for generation of graphs. All quantitative data are presented as median values with minimal to maximal range. The analysis was performed using two-sided Fisher’s exact test for categorical data and two-sided Mann–Whitney *U* test with continuity correction for quantitative data. Logistic regression analysis was adjusted for age and sex. Allele distribution in European population was retrieved from 1000 Genomes Project Phase 3 database (www.internationalgenome.org). Chi-square test was used for comparison with the results. Two-sided sign test for matched pairs was applied to compare the same parameters between different time points. *P* values less than 0.05 were considered statistically significant.

## Results

Selected characteristics of individual patients during and after DAA therapy are shown in Table S2. All 42 subjects were HCV-RNA negative in serum 24 weeks and remained negative 12–15 months after EOT. Thirty-one patients (74%) were positive for HCV-RNA in PBMCs, and in 29 (69%) of them we confirmed the presence of HCV-RNA (−) strand. Normalization of ALT at the follow-up was noted in 34 individuals (81%). Neither ALT level nor ALT normalization after therapy correlated with the presence of HCV-RNA (Tables 1, S2; Fig. S2). Treatment with OBV/PTV/r + DSV ± RBV was most effective in eradication of replicative viral RNA strand (Table 1).

**Table 1:** Characteristics of groups of patients with or without HCV-RNA (−) strand in PBMCs

Characteristics	HCV-RNA (−) strand in PBMC		*P* value^a^
	Present (*n* = 29)	Absent (*n* = 13)	
Age	55 (29–66)	59 (39–70)	0.136
Sex (female)	14 (48%)	7 (54%)	1.000
Liver cirrhosis^b^	20 (69%)	10 (77%)	0.722
HCC^b^	3 (10%)	0	0.540
Cryoglobulinemia^b^	6 (21%)	3 (23%)	1.000
Treatment naïve	6 (21%)	4 (31%)	0.697
Duration of CHC (years)^c^	11 (0.5–18)	12 (2–19)	0.851
HCV-RNA (kIU/mL)^b^	817 (3.1–17,650)	1027.5 (13.2–37,900)	0.988
DAA treatment			
OBV/PTV/r + DSV ± RBV	12 (41%)	11 (85%)	**0.017**
SOF/LDV + RBV	15 (52%)	2 (15%)	**0.041**
SOF + RBV	2 (7%)	0	0.471
IFNλ3 rs12979860 genotype			
CC	4 (14%)	4 (31%)	0.226
TT	8 (28%)	1 (8%)	0.231
CT	17 (59%)	8 (62%)	0.567
Adverse events^d^	7 (24%)	3 (23%)	1.000
Normalization of ALT at follow-up	22 (76%)	12 (92%)	0.398
Δ neutrocyte count (EOT–baseline) [× 10^9^ cells/L]	0.03 (− 1.22–4.64)	0.69 (− 0.57–3.32)	**0.028**
Δ lymphocyte count (EOT–baseline) [× 10^9^ cells/L]	− 0.19 (− 1.42–1.05)	− 0.32 (− 0.81–0.23)	0.374

Significant *P* values < 0.05 are given in bold

*ALT* alanine aminotransferase; *CHC* chronic hepatitis C; *EOT* end of treatment; *OBV/PTV/r+DSV±RBV* ombitasvir, paritaprevir, ritonavir, dasabuvir, ribavirin; *SOF*/*LDV* + *RBV* sofosbuvir, ledipasvir, ribavirin; *SOF* + *RBV* sofosbuvir, ribavirin. For quantitative data median values with minimal maximal range are given

^a^For differences between groups with and without HCV-RNA (−) in PBMCs, two-sided Fisher’s exact test for categorical data, two-sided Mann–Whitney *U* test with continuity correction for quantitative data

^b^Before DAA therapy

^c^From the diagnosis of CHC to the start of DAA treatment

^d^Adverse events during therapy and follow-up

In case of 10 patients adverse events occurred during DAA therapy and follow-up (Table S2), including portal vein thrombosis with liver failure, ascites, severe bacterial infections, vasculitis, nephropathy, monoclonal gammopathy, lymphomas and rapidly elevating ALT after treatment (Table S2). In three cases of hepatocellular carcinoma (HCC) diagnosed and treated with radiofrequency ablation before therapy no recurrence was observed during follow-up. Occurrence of adverse events did not correlate with the presence of HCV-RNA in PBMCs (Table 1, Table S3), but it associated with lower lymphocyte counts during and after therapy (Fig. S3).

Polymorphisms rs368234815 ΔG/T and rs12979860 T/C were in a complete linkage disequilibrium (*r*2 = 1), so further we will refer only to rs12979860. Allele frequencies for rs12979860 (*f*_C_ = 0.488; *f*_T_ = 0.512) differed significantly from their distribution in European population (*f*_C_ = 0.691; *f*_T_ = 0.309), with unfavorable, minor T allele being more frequent than in population (Chi^2^ = 16.197; *P* < 0.0001). There was no correlation between *IFNL3* genotype and the presence of HCV-RNA in PBMCs (Table 1; Table S3). Favorable homozygote rs12979860 CC had lower neutrocyte levels at the beginning of therapy (2.2 vs. 3.2 × 10^9^ cells/L; *P* = 0.024) and after 24 weeks (2.3 vs. 3.3 × 10^9^ cells/L; *P* = 0.037) (Fig. S4).

Test for matched pairs revealed that for all patients significant fall of lymphocyte number occurred during therapy [1.75 × 10^9^ (0.61–4.5) vs. 1.32 × 10^9^ (0.42–5.36) at EOT; *P* = 0.009)], and neutrophils remained at the same level (*P* = 0.090) (Fig. S5A–B). During 12 weeks of therapy NLR, SII and PLR values rose significantly for all individuals (*P* < 0.0001 for all markers), while MPV decreased (*P* = 0.001) (Fig. S5C–F). Additionally, SII and PLR remained significantly higher from baseline at the follow-up (*P* = 0.02 and 0.044, respectively) (Fig. S5D–E).

**Fig. 1:**
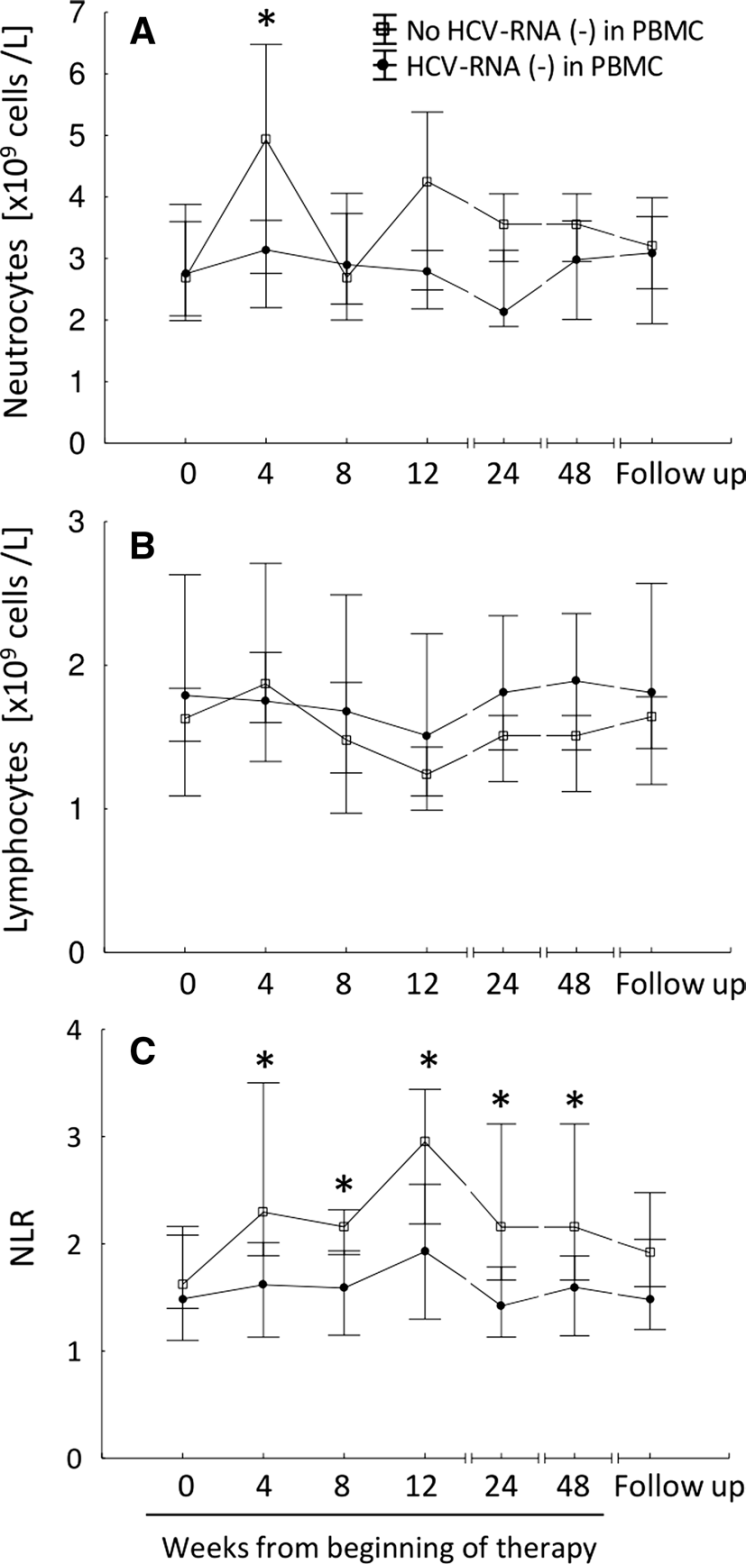
Changes in neutrocyte and lymphocyte counts and NLR values for groups of patients with or without HCV-RNA (−) strand. Neutrocyte (**a**) and lymphocyte (**b**) counts as well as NLR values (**c**) were recorded during 12 weeks of DAA treatment and up to 60–72 weeks (follow-up) after the start of therapy. Points show median values; whiskers represent percentiles (25th to 75th). Filled circles, patients with HCV-RNA (−) strand in PBMCs; empty squares, patients without HCV-RNA (−) strand detected in PBMCs. NLR, neutrocyte-to-lymphocyte ratio; *, significant (*P* < 0.05) differences between groups of patients in a given time point calculated with two-sided Mann–Whitney *U* test

There were no significant differences in neutrocyte and lymphocyte counts between groups of patients with or without viral RNA in PBMCs (Fig. 1a, b). However, a smaller increase in neutrocyte count between EOT and baseline was observed in patients with HCV-RNA (−) (Table 1). Higher NLR value during and shortly after therapy associated with clearance of replicative HCV-RNA strand (Table 2; Fig. 1c). Among patients without HCV-RNA (−) NLR was significantly higher from baseline at 4, 8, 12 weeks after the start of therapy (*P* values from sign test 0.027, 0.006 and 0.006, respectively), while in the group with replicative strand NLR was significantly raised from baseline only after 12 weeks (*P* = 0.001) (Fig. 1c). Also lower SII linked with detected HCV-RNA (−) in PBMC but only at 4 and 24 weeks after the start of therapy (Table S4; Fig. S6A). Association of NLR and SII with the presence of total HCV-RNA in PBMCs was much weaker (Table S2, Table S4). There were no significant differences in PLR or MPV between groups of patients (Table S4; Fig. S6B–C).

**Table 2:** NLR values in groups of patients with or without HCV-RNA (−) strand in PBMCs

Characteristics	HCV-RNA (−) strand in PBMC		*P* value^a^	OR (CI 95%)^b^	*P* value^b^
	Present (*n* = 29)	Absent (*n* = 13)			
0-week NLR	1.5 (0.5–7.5)	1.6 (0.8–6.3)	0.305	0.80 (0.51–1.27)	0.330
4-week NLR	1.6 (0.6–5.4)	2.3 (1.0–59.0)	**0.012**	0.68 (0.36–1.30)	0.230
8-week NLR	1.6 (0.8–6.2)	2.2 (1.4–8.4)	**0.011**	0.69 (0.41–1.17)	0.156
12-week NLR (EOT)	1.9 (0.6–3.7)	3.0 (1.3–9.3)	**0.017**	0.65 (0.40–1.06)	0.077
24-week NLR	1.4 (0.5–3.7)	2.2 (1.5–4.6)	**0.002**	0.23 (0.10–0.86)	**0.019**
48-week NLR (SVR24)	1.6 (0.5–5.7)	2.2 (1.4–4.6)	**0.021**	0.65 (0.34–1.23)	0.172
60–72-week NLR (follow-up)	1.5 (0.7–5.7)	1.9 (0.8–4.6)	0.185	0.87 (0.47–1.62)	0.647

Median NLR values with minimal to maximal range are given

Significant *P* values < 0.05 are given in bold

*EOT* end of treatment; *NLR* neutrocyte-to-lymphocyte ratio; *OR* odds ratio; *CI* confidence intervals

^a^For difference between groups with and without HCV-RNA (−), two-sided Mann–Whitney *U* test with continuity correction

^b^Logistic regression analysis adjusted for age and sex

## Discussion

This brief report shows high occurrence of occult HCV infection in patients who reached SVR24 after DAAs. In the majority of these subjects we also found a replicative HCV-RNA (−) strand. These results are consistent with observations of Elmasry et al. that while in the liver of CHC patients a 1–3 logs difference in the number of copies is seen in favor of HCV-RNA (+) strand, in OCI the amounts of HCV-RNA (+) and (−) strand are similar [10].

Normalization of ALT was not a predictor of OCI or the presence of HCV-RNA (−). This is in accordance with recent report in which elevated liver enzymes after DAA therapy did not predict persistence of viral RNA [10]. Additionally, OCI has been frequently detected in patients after successful IFN-based therapy having normal levels of liver enzymes [13, 14].

It is now established that genetic variations within *IFNL3*-*IFNL4* gene region impact interferon-stimulated gene expression (ISGs) both in the liver and in PBMCs of CHC patients and are predictors of spontaneous and treatment-induced HCV clearance in both DAA- and IFN-based therapy [15–17]. However, their impact on development of OCI is still unclear. Elmasry et al. [10] examined 9 liver transplant recipients who did not reach normalization of liver enzymes after DAA therapy. They found OCI in all homozygotes in unfavorable rs12979860 T allele (*n* = 5), while carriers of C/T and CC alleles did not have HCV-RNA in liver tissue or PBMCs [10]. In another study prevalence of the rs12979860 CC genotype was significantly higher in patients with seronegative OCI (no anti-HCV Abs) than in CHC group. Additionally, among patients with this type of occult infection the presence of rs12979860 CC genotype associated with lower HCV-RNA load in liver tissue [18]. Polymorphisms in the *IFNL3*-*IFNL4* region also link with the presence of HCV-RNA in PBMCs both during chronic HCV infection and in patients who reached SVR after IFN treatment [19, 20]. We found no significant correlation between *IFNL* genotype and the occurrence of HCV-RNA in PBMCs. This may be due to a small group of patients which is not representative of a whole population, as the unfavorable rs12979860 T allele is significantly more prevalent in the tested group. It was shown that at the beginning of DAA therapy rs12979860 CC carriers exhibit decreased expression of ISGs in peripheral blood and liver [17]. As ISGs represent a diverse group of gene products which can also act as inflammatory cytokines this observation corresponds to lower neutrocyte levels at baseline observed in our study for rs12979860 C homozygotes.

We aimed to evaluate if markers of systemic inflammation, such as NLR, SII, PLR and MPV, which are useful prognostic factors in many chronic diseases, can predict development of OCI after DAAs. It was reported that increased PLR in CHC patients during therapy predicts a good virological response [21]. MPV indicates inflammation and liver damage in CHC patients [22], while SII is a prognostic factor in patients with HCC [23].

Therapy with DAAs is known to evoke dynamic changes in cytokine profile and in the balance between neutrophil and lymphocyte counts, as well as to induce a general inflammatory state [23–25]. In DAA-treated patients a significantly greater increase in neutrophil counts and decrease in lymphocyte numbers between EOT and baseline associated with later development of HCC after therapy [25]. Viral clearance and SVR induced by DAA therapy is accompanied with downregulation of signaling with type II and type III IFNs, but also with elevation of IFN I cellular pathways, which can promote elimination of the virus [26]. In patients achieving SVR after DAAs ISGs expression in liver and blood at EOT is significantly increased in comparison with baseline [27].

We found that a significant and transient rise in NLR, a marker of systemic inflammation, during and shortly after DAA therapy can predict the clearance of replicating HCV from PBMCs. Additionally, patients without HCV-RNA (−) strand had a significantly greater increase in neutrocyte counts between EOT and baseline. We hypothesize that differences in patient’s immune responsiveness at baseline and in the ability to induce general inflammatory response after viral suppression by DAAs determine subsequent HCV persistence/clearance. Although this systemic inflammation could be beneficial for complete eradication of HCV, it might also favor carcinogenesis [23, 25].

Our study is limited by a small sample size and restricted to patients infected with HCV genotype 1. Therefore, further research on larger cohorts is needed to find if transient rise in NLR could be used as a predictor of viral clearance. Also long-term follow-up studies of patients with residual HCV-RNA should be performed to examine the natural course of secondary occult infection after DAAs and its clinical significance.

## Electronic supplementary material

Below is the link to the electronic supplementary material.

Supplementary material 1 (DOCX 569 kb)

## References

[1] Pham TN, Michalak TI (2011). Occult hepatitis C virus infection and its relevance in clinical practice. J Clin Exp Hepatol.

[2] Chen CL, Huang JY, Wang CH (2017). Hepatitis C virus has a genetically determined lymphotropism through co-receptor B7.2. Nat Commun.

[3] Skardasi G, Chen AY, Michalak TI (2018). Authentic patient-derived hepatitis C virus infects and productively replicates in primary CD4 + and CD8 + T lymphocytes in vitro. J Virol.

[4] Kondo Y, Shimosegawa T (2013). Direct effects of hepatitis C virus on the lymphoid cells. World J Gastroenterol.

[5] Dai B, Chen AY, Corkum CP (2016). Hepatitis C virus upregulates B-cell receptor signaling: a novel mechanism for HCV-associated B-cell lymphoproliferative disorders. Oncogene.

[6] Roque-Cuéllar MC, Sánchez B, García-Lozano JR (2014). Hepatitis C virus-specific cellular immune responses in sustained virological responders with viral persistence in peripheral blood mononuclear cells. Liver Int.

[7] Radkowski M, Opoka-Kegler J, Cortes KC (2014). Evidence for immune activation in patients with residual hepatitis C virus RNA long after successful treatment with IFN and ribavirin. J Gen Virol.

[8] Attar BM, Van Thiel DA (2015). New twist to a chronic HCV infection: occult hepatitis C. Gastroenterol Res Pract.

[9] Gambato M, Pérez-del-pulgar S, Hedskog C (2016). Hepatitis C virus RNA persists in liver explants of most patients awaiting liver transplantation treated with an interferon-free regimen. Gastroenterology.

[10] Elmasry S, Wadhwa S, Bang B-R (2017). Detection of occult hepatitis C virus infection in patients who achieved a sustained virologic response to direct-acting antiviral agents for recurrent infection after liver transplantation. Gastroenterology.

[11] Wróblewska A, Bernat A, Woziwodzka A (2017). Interferon lambda polymorphisms associate with body iron indices and hepatic expression of interferon-responsive long non-coding RNA in chronic hepatitis C. Clin Exp Med.

[12] Kato T, Date T, Miyamoto M (2003). Efficient replication of the genotype 2a hepatitis C virus subgenomic replicon. Gastroenterology.

[13] Pham TNQ, MacParland SA, Mulrooney PM, Cooksley H, Naoumov NV, Michalak TI (2004). Hepatitis C virus persistence after spontaneous or treatment-induced resolution of hepatitis C. J Virol.

[14] Radkowski M, Horban A, Gallegos-Orozco JF (2005). Evidence for viral persistence in patients who test positive for anti-hepatitis C virus antibodies and have normal alanine aminotransferase levels. J Infect Dis.

[15] Prokunina-Olsson L, Muchmore B, Tang W (2013). A variant upstream of IFNL3 (IL28B) creating a novel interferon gene IFNL4 is associated with impaired clearance of hepatitis C virus. Nat Genet.

[16] Rosenberg BR, Freije CA, Imanaka N (2017). Genetic variation at IFNL4 influences extrahepatic interferon-stimulated gene expression in chronic HCV patients. J Infect Dis.

[17] Ramamurthy N, Marchi E, Ansari MA (2018). Impact of IFNL4 genotype on interferon-stimulated gene expression during DAA therapy for hepatitis C. Hepatology.

[18] Bartolomé J, Castillo I, Quiroga JA, Carreño V (2016). Interleukin-28B polymorphisms and interferon gamma inducible protein-10 serum levels in seronegative occult hepatitis C virus infection. J Med Virol.

[19] Miri SM, Sharafi H, Pouryasin A (2017). The role of polymorphisms Near IFNL3 gene as predictors of residual HCV RNA in buffy coat after successful antiviral therapy. Hepat Mon.

[20] Angulo J, Pino K, Pavez C (2013). Genetic variations in host IL28B links to the detection of peripheral blood mononuclear cells–associated hepatitis C virus RNA in chronically infected patients. J Viral Hepat.

[21] Meng X, Wei G, Chang Q (2016). The platelet-to-lymphocyte ratio, superior to the neutrophil-to-lymphocyte ratio, correlates with hepatitis C virus infection. Int J Infect Dis.

[22] Purnak T, Olmez S, Torun S (2013). Mean platelet volume is increased in chronic hepatitis C patients with advanced fibrosis. Clin Res Hepatol Gastroenterol.

[23] Casadei Gardini A, Foschi FG, Conti F, Petracci E, Vukotic R, Marisi G (2018). Immune inflammation indicators and ALBI score to predict liver cancer in HCV-patients treated with direct-acting antivirals. Dig Liver Dis.

[24] Debes JD, van Tilborg M, Groothuismink ZMA (2018). Levels of cytokines in serum associate with development of hepatocellular carcinoma in patients with HCV infection treated with direct-acting antivirals. Gastroenterology.

[25] Casadei Gardini A, Conti A, Brillanti F (2018). Imbalance of neutrophils and lymphocyte counts can be predictive of hepatocellular carcinoma occurrence in hepatitis C-related cirrhosis treated with direct-acting antivirals. Gastroenterology.

[26] Meissner EG, Wu D, Osinusi A (2014). Endogenous intrahepatic IFNs and association with IFN-free HCV treatment outcome. J Clin Invest.

[27] Alao H, Cam M, Keembiyehetty C (2018). Baseline intrahepatic and peripheral innate immunity are associated with hepatitis C virus clearance during direct-acting antiviral therapy. Hepatology.

