# Supplementary material for: Neutrocyte-to-lymphocyte ratio predicts the presence of a replicative hepatitis C virus strand after therapy with direct-acting antivirals
Source: Clin Exp Med. 2019 May 24;19(3):401–6. doi: 10.1007/s10238-019-00561-y (PMC6647462; doi:10.1007/s10238-019-00561-y)
Supplement: Supplementary file 1 — Supplementary material 1 (DOCX 569 kb) [file 10238_2019_561_MOESM1_ESM.docx]

Clinical and Experimental Medicine

Neutrocyte to lymphocyte ratio predicts the presence of a replicative hepatitis C virus strand after therapy with directly acting antivirals

Anna Wróblewska, Beata Lorenc, Małgorzata Cheba, Krzysztof P. Bielawski, Katarzyna Sikorska

**Correspondence:**

Katarzyna Sikorska MD, PhD

Department of Tropical Medicine and Epidemiology, Medical University of Gdansk, Poland, Powstania Styczniowego 9b, 81-519, Gdynia

Tel./fax: +48 58 349 17 60; Email: ksikorska@gumed.edu.pl

**Table of contents**

**Patients and methods ...............................................................................................................3**

Isolation and stimulation of PBMCs ..........................................................................................3

Ultrasensitive detection of HCV-RNA ......................................................................................3

**Results........................................................................................................................................5**

**Fig. S1** Exemplary results of HCV-RNA (-) strand detection in PBMC samples......................**5**

**Table S1** Selected baseline characteristics of all patients enrolled in the study.........................**6**

**Table S2** Selected characteristics of individual patients............................................................**7**

**Fig. S2** Changes in ALT levels in groups of patients with or without HCV-RNA (-) strand...............................................................................................................................**11**

**Fig. S3** Changes in lymphocyte counts in groups of patients with or without adverse events...............................................................................................................................**12**

**Fig. S4** Changes in neutrocyte counts in groups of patients with different IFNL genotypes...**13**

**Fig. S5** Time-course changes in neutrocyte and lymphocyte counts and values of inflammation markers for all patients..............................................................................**14**

**Fig. S6** Changes in SII, PLR and MPV values in groups of patients with or without HCV-RNA (-) strand.................................................................................................................**15**

**Table S3** Comparison of groups of patients with or without total HCV-RNA in PBMCs......**16**

**Table S4** Comparison of MPV, PLR and SII values between groups of patients with or without HCV-RNA in PBMCs........................................................................................**17**

**Isolation and stimulation of peripheral blood mononuclear cells (PBMCs)**

PBMCs were isolated using Histopaque (Sigma) gradient centrifugation and incubated for 72h in RPMI 1640 with 10% FBS and 1% penicillin-streptomycin-glutamine (Gibco) in the presence of mitogens: 10 μg/mL phytohemagglutinin-M (Roche Applied Science, Germany) and 5 μg/mL concanavalin A (Sigma). Cells were then collected and stored in -80°C.

**Ultrasensitive detection of HCV-RNA**

Total RNA was isolated using TriReagent (Ambion, USA) according to manufacturer instructions. RNA grade glycogen (Thermo Fisher, USA) was added to the final concentration 0.05 μg/mL at precipitation step. For total HCV-RNA detection RNA was reverse transcribed with RevertAid Kit (Thermo Fisher, USA) using random hexamers as primers. For detection of HCV-RNA negative strand RNA was transcribed with Tth polymerase (Promega, Germany) and forward Tth_F primer 5’-GTCTTCACGCAGAAAGCGTCTAGCCATGG-3’ followed by chelation of manganese ions. The procedure was performed according to manufacturer instructions, with modifications. Briefly, for a 10μl reaction 1 mM MnCl_2_, 1 x reverse transcriptase (RT) buffer, 0.2 mM dNTPs mixture, 0.75 μM of primer, 2U Tth polymerase and 1.1 μg of total RNA was added. The mixture was incubated at 70°C for 20 minutes. The reaction was stopped by adding EGTA to the final concentration of 1.5 mM.

Final HCV-RNA detection of both total and negative strand of HCV-RNA was performed with real time PCR using LightCycler 480 SYBR Green I Master and LightCycler 480 (Roche Applied Science, Germany), and with the following primers: forward 5’UTR_F 5’-GTCTTCACGCAGAAAGCGTC-3’, and reverse 5’UTR_R 5’-CCTGGCAATTCCGGTGTACT-3’, amplifying a fragment of 5’ untranslated region of HCV genome. The PCR reaction was carried for 45 cycles with primer annealing temperature at 60°C. Each cDNA sample was amplified at least in 2 real time PCR reactions, each 50 μl. Every reaction contained cDNA synthesized from at least 500ng of total RNA. Maximal volume of RT reaction added for 50 μl of final real time PCR reaction was 5 μl.

During all the procedures both positive and negative controls were processed in parallel with patients samples. As positive control samples we used PBMCs and liver biopsy from chronic hepatitis C (CHC) patient. Samples of mitogen stimulated PBMCs from two healthy volunteers and liver biopsy from HCV-RNA negative patient with non-alcoholic fatty liver disease (NAFLD) were used as negative controls. Specificity of obtained PCR products was confirmed by melting curve analysis and sequencing. Exemplary results of HCV-RNA (-) strand detection are presented in Fig. S1.

**Fig. S1** Exemplary results of HCV-RNA (-) strand detection in PBMC samples

Amplification curves (**a**) and melting peaks (**b**) from the final real time PCR step are presented. Products of the amplification were separated in a 2% agarose gel (**c**). A 122 base pair (bp) product is shown with arrow. P1-P3, positive patients samples; P4-P6, negative patients samples; C1, negative control – PBMC sample from healthy volunteer; C2, negative control – liver biopsy sample from Anti-HCV-Ab (-) NAFLD patient; C3, positive control - PBMC sample from CHC patient; C4, positive control – liver biopsy sample from CHC patient; N1, - no template control of RT step with Tth polymerase; N2 - no template control of real time PCR; M, 100bp DNA marker.

**Table S1** Selected baseline characteristics of all patients enrolled in the study

| Characteristics | | | All patients (n=42) |
| --- | --- | --- | --- |
|  |  |  |  |
| Age | | | 57.5 (29-70) |
| Sex (Female) | | | 21 (50%) |
| Liver cirrhosis | | | 30 (71%) |
| HCC | | | 3 (7%) |
| Cryoglobulinemia | | | 9 (21%) |
| Treatment naiive | | | 10 (24%) |
| Duration of CHC [years] | | | 11 (0.5-19) |
| HCV-RNA [kIU/mL] | | | 901 (3.1-17650) |
| ALT [IU/L] | | | 78 (27-272) |
| Neutrocytes [×10^9^/L] | | | 2.8 (0.8-8.2) |
| Lymphocytes [×10^9^/L] | | | 1.7 (0.6-4.5) |
| Platelets [×10^9^/L] | | | 104 (45-351) |
| DAA treatment | OBV/PTV/r+DSV±RBV | | 23 (55%) |
|  | SOF/LDV+RBV | | 17 (41%) |
|  | SOF+RBV | | 2 (5%) |
| IFNλ3 rs12979860 genotype | | CC | 8 (19%) |
|  |  | TT | 9 (21%) |
|  |  | CT | 25 (60%) |

ALT, alanine transaminase; CHC, chronic hepatitis C; OBV/PTV/r+DSV±RBV, ombitasvir, paritaprevir, ritonavir, dasabuvir, ribavirin; SOF/LDV+RBV, sofosbuvir, ledipasvir, ribavirin; SOF+RBV, sofosbuvir, ribavirin. For quantitative data median values with minimal maximal range are given.

**Table S2** Selected characteristics of individual patients

| **Sex** | **Age** | ***IFNL*^a^** | **History^b^** | **CHC [yrs]** | **Follow up [wks]** | **DAA^c^** | **HCV-RNA in PBMC^d^** | |  | **Clinical** | | |  | **Lymphocytes [×10^9^/L]** | | | **Neutrocytes [×10^9^/L]** | | | **Platelets [×10^9^/L]** | | | **ALT [IU/L]** | | | **NLR** | | |
| --- | --- | --- | --- | --- | --- | --- | --- | --- | --- | --- | --- | --- | --- | --- | --- | --- | --- | --- | --- | --- | --- | --- | --- | --- | --- | --- | --- | --- |
|  |  |  |  |  |  |  | **Total** | **(-)** |  | **Before DAA^e^** | **Adverse**  **events^f^** | |  | **0** | **EOT** | **F** | **0** | **EOT** | **F** | **0** | **EOT** | **F** | **0** | **EOT** | **F** | **0** | **EOT** | **F** |
| K | 59 | CT | TN | 10 | 68 | SL+R | 0 | 0 |  | LC, CRYO |  | |  | 1.6 | 1.3 | 1.4 | 2.3 | 4.3 | 3.0 | 93 | 117 | 111 | 49 | 19 | 11 | 1.4 | 3.3 | 2.1 |
| K | 59 | CC | R | 8 | 67 | OPD+R | 0 | 0 |  | LC |  | |  | 1.8 | 1.0 | 1.7 | 2.1 | 3.3 | 3.2 | 87 | 118 | 111 | 98 | 17 | 15 | 1.2 | 3.4 | 1.9 |
| M | 58 | CT | N | 14 | 67 | OPD+R | 0 | 0 |  | LC |  | |  | 2.0 | 1.8 | 1.6 | 3.9 | 5.4 | 3.2 | 135 | 195 | 119 | 72 | 47 | 66 | 5.4 | 9.3 | 4.6 |
| M | 44 | CT | N | 19 | 68 | OPD+R | 0 | 0 |  | LC |  | |  | 1.8 | 1.4 | 1.8 | 3.8 | 4.7 | 4.4 | 181 | 238 | 243 | 87 | 23 | 31 | 1.1 | 1.7 | 1.5 |
| M | 58 | CT | R | 12 | 65 | OPD+R | 0 | 0 |  | LC |  | |  | 1.9 | 1.8 | 1.9 | 1.6 | 2.3 | 1.5 | 138 | 237 | 159 | 61 | 29 | 33 | 1.2 | 1.8 | 1.9 |
| M | 66 | TT | N | 18 | 68 | OPD+R | 0 | 0 |  |  |  | |  | 1.5 | 0.9 | 1.5 | 2.7 | 2.1 | 2.5 | 80 | 80 | 87 | 186 | 27 | 22 | 0.8 | 1.4 | 1.6 |
| K | 56 | CT | TN | 2 | 65 | OPD+R | 0 | 0 |  | LC, CRYO | during DAA severe bacterial infection with ascites | |  | 0.6 | 0.7 | 0.7 | 3.4 | 6.7 | 3.2 | 289 | 493 | 341 | 76 | 11 | 13 | 1.1 | 1.6 | 1.4 |
| M | 39 | CT | TN | 13 | 62 | SL+R | 0 | 0 |  | LC | bleeding of esophageal varices severe bacterial infection of urinary tract after treatment | |  | 1.6 | 1.2 | 1.8 | 2.2 | 2.7 | 2.7 | 45 | 50 | 47 | 99 | 27 | 25 | 2.5 | 2.0 | 4.6 |
| K | 70 | TT | I | 6 | 68 | OPD+R | 0 | 0 |  | LC |  | |  | 1.3 | 1.2 | 1.0 | 2.1 | 2.5 | 1.6 | 59 | 78 | 66 | 27 | 26 | 16 | 1.1 | 1.5 | 1.5 |
| K | 60 | CT | R | 5 | 68 | OPD+R | 0 | 0 |  |  |  | |  | 2.6 | 1.9 | 2.5 | 3.9 | 4.5 | 4.0 | 294 | 364 | 307 | 29 | 13 | 14 | 7.6 | 3.5 | 1.3 |
| M | 66 | CT | R | 4 | 68 | OPD+R | 0 | 0 |  |  |  | |  | 1.8 | 1.2 | 1.7 | 2.0 | 2.1 | 1.9 | 120 | 145 | 131 | 27 | 15 | 22 | 4.6 | 6.2 | 3.6 |
| K | 70 | CT | TN | 17 | 68 | OPD+R | 1 | 0 |  | LC, CRYO |  | |  | 1.5 | 1.3 | 1.5 | 5.2 | 5.4 | 4.4 | 211 | 266 | 210 | 77 | 17 | 32 | 2.1 | 3.3 | 2.5 |
| K | 51 | TT | N | 12 | 65 | OPD+R | 1 | 0 |  | LC | nephropathy after DAA | |  | 0.9 | 1.1 | 1.0 | 5.5 | 6.2 | 3.7 | 87 | 91 | 109 | 70 | 37 | 15 | 1.5 | 1.1 | 1.1 |
| **Sex** | **Age** | ***IFNL*** | **History** | **CHC [yrs]** | **Follow up [wks]** | **DAA** | **HCV-RNA in PBMC** | |  | **Clinical** | | |  | **Lymphocytes [×10^9^/L]** | | | **Neutrocytes [×10^9^/L]** | | | **Platelets [×10^9^/L]** | | | **ALT [IU/L]** | | | **NLR** | | |
|  |  |  |  |  |  |  | **Total** | **(-)** |  | **Before DAA** | | **Adverse**  **events** |  | **0** | **EOT** | **F** | **0** | **EOT** | **F** | **0** | **EOT** | **F** | **0** | **EOT** | **F** | **0** | **EOT** | **F** |
| K | 47 | CT | R | 17 | 67 | OPD | 1 | 1 |  |  | | lymphoma after treatment |  | 4.5 | 5.4 | 4.1 | 3.6 | 3.1 | 6.1 | 224 | 239 | 263 | 42 | 21 | 20 | 1.9 | 3.0 | 2.0 |
| M | 55 | CC | N | 13 | 67 | OPD+R | 1 | 1 |  | LC | |  |  | 2.8 | 2.2 | 2.3 | 3.1 | 3.7 | 3.4 | 145 | 187 | 167 | 91 | 17 | 54 | 0.8 | 1.3 | 0.8 |
| M | 58 | CT | R | 17 | 67 | OPD+R | 1 | 1 |  | LC | |  |  | 1.7 | 1.5 | 3.4 | 2.8 | 2.9 | 4.0 | 121 | 150 | 171 | 101 | 88 | 19 | 1.8 | 2.5 | 1.7 |
| M | 60 | TT | TN | 1 | 68 | OPD+R | 1 | 1 |  | LC, HCC | |  |  | 3.9 | 3.9 | 4.5 | 3.3 | 4.4 | 5.9 | 351 | 382 | 369 | 75 | 16 | 22 | 1.4 | 2.2 | 1.5 |
| K | 29 | CC | N | 6 | 61 | S+R | 1 | 1 |  | LC | | rapid ALT rise after treatment |  | 1.0 | 1.2 | 1.0 | 1.3 | 2.0 | 1.8 | 63 | 70 | 77 | 161 | 18 | 62 | 1.6 | 2.0 | 1.7 |
| K | 35 | CT | N | 10 | 61 | SL+R | 1 | 1 |  | LC | |  |  | 3.6 | 2.2 | 3.2 | 2.8 | 3.1 | 5.1 | 199 | 224 | 213 | 82 | 24 | 23 | 1.5 | 2.4 | 1.6 |
| M | 38 | CC | N | 6 | 62 | S+R | 1 | 1 |  | LC | | rapid ALT rise after treatment |  | 1.8 | 1.1 | 1.5 | 2.0 | 1.7 | 2.1 | 95 | 93 | 122 | 91 | 56 | 110 | 1.1 | 1.8 | 1.1 |
| M | 62 | CT | TN | 16 | 61 | SL+R | 1 | 1 |  | HCC | |  |  | 1.4 | 1.0 | 2.0 | 2.8 | 2.5 | 3.5 | 247 | 223 | 209 | 116 | 21 | 25 | 3.5 | 4.2 | 2.9 |
| M | 66 | CT | TN | 17 | 61 | SL+R | 1 | 1 |  | LC | |  |  | 0.8 | 0.8 | 1.7 | 1.3 | 1.6 | 2.3 | 68 | 87 | 97 | 57 | 28 | 33 | 6.3 | 5.7 | 3.5 |
| K | 59 | TT | I | 13 | 61 | SL+R | 1 | 1 |  | LC, CRYO | |  |  | 1.1 | 0.7 | 0.8 | 0.8 | 0.8 | 0.8 | 51 | 58 | 50 | 81 | 22 | 21 | 0.8 | 0.6 | 1.5 |
| K | 53 | CT | N | 15 | 72 | OPD+R | 1 | 1 |  | LC | | portal vein thrombosis, liver failure, ascites, encephalopathy and pleural effusion during therapy |  | 0.7 | 0.9 | 0.3 | 1.8 | 1.8 | 1.6 | 83 | 70 | 70 | 132 | 34 | 28 | 1.6 | 1.9 | 1.2 |
| K | 44 | CT | N | 7 | 64 | SL+R | 1 | 1 |  | LC | |  |  | 1.0 | 0.8 | 0.9 | 1.6 | 2.2 | 1.2 | 46 | 49 | 42 | 27 | 21 | 14 | 0.8 | 1.1 | 1.3 |
| K | 62 | CT | N | 15 | 61 | SL+R | 1 | 1 |  | CRYO | |  |  | 2.1 | 2.2 | 1.4 | 2.2 | 2.8 | 3.5 | 203 | 212 | 221 | 28 | 11 | 11 | 2.0 | 2.6 | 1.8 |
| M | 54 | CC | R | 12 | 68 | OPD+R | 1 | 1 |  | CRYO | |  |  | 3.7 | 2.4 | 2.4 | 4.0 | 3.6 | 3.6 | 191 | 295 | 295 | 124 | 35 | 35 | 1.6 | 1.9 | 1.3 |
| K | 62 | CT | N | 8 | 61 | SL+R | 1 | 1 |  |  | |  |  | 1.2 | 0.8 | 1.0 | 2.7 | 2.0 | 1.9 | 86 | 70 | 61 | 51 | 32 | 22 | 0.7 | 1.2 | 1.0 |
| **Sex** | **Age** | ***IFNL*** | **History** | **CHC [yrs]** | **Follow up [wks]** | **DAA** | **HCV-RNA in PBMC** | |  | **Clinical** | | |  | **Lymphocytes [×10^9^/L]** | | | **Neutrocytes [×10^9^/L]** | | | **Platelets [×10^9^/L]** | | | **ALT [IU/L]** | | | **NLR** | | |
|  |  |  |  |  |  |  | **Total** | **(-)** |  | **Before DAA** | **Adverse**  **events** | |  | **0** | **EOT** | **F** | **0** | **EOT** | **F** | **0** | **EOT** | **F** | **0** | **EOT** | **F** | **0** | **EOT** | **F** |
| K | 62 | CT | TN | 18 | 62 | SL+R | 1 | 1 |  | LC |  | |  | 2.7 | 2.1 | 2.8 | 1.4 | 2.2 | 1.9 | 138 | 136 | 115 | 126 | 10 | 13 | 1.6 | 2.8 | 1.3 |
| M | 57 | TT | N | 9 | 64 | SL+R | 1 | 1 |  | LC |  | |  | 1.2 | 1.2 | 1.8 | 2.0 | 2.4 | 2.1 | 91 | 109 | 113 | 152 | 49 | 60 | 1.0 | 1.3 | 2.5 |
| K | 33 | CT | TN | 2 | 62 | SL+R | 1 | 1 |  |  |  | |  | 1.1 | 2.1 | 1.5 | 8.2 | 7.4 | 1.9 | 335 | 405 | 274 | 42 | 24 | 38 | 2.2 | 2.5 | 2.0 |
| M | 63 | CT | R | 12 | 61 | SL+R | 1 | 1 |  |  | nephropathy after DAA | |  | 0.6 | 0.5 | 0.8 | 3.0 | 2.8 | 4.5 | 83 | 109 | 149 | 272 | 27 | 18 | 0.5 | 1.0 | 0.7 |
| M | 60 | CC | R | 9 | 68 | OPD+R | 1 | 1 |  |  |  | |  | 1.8 | 2.2 | 2.6 | 3.8 | 8.5 | 2.3 | 133 | 193 | 160 | 254 | 31 | 15 | 1.6 | 2.0 | 1.1 |
| K | 37 | TT | N | 6 | 68 | OPD+R | 1 | 1 |  | LC |  | |  | 2.7 | 2.5 | 3.5 | 3.0 | 3.1 | 4.0 | 102 | 111 | 119 | 117 | 48 | 48 | 4.7 | 5.4 | 5.7 |
| M | 53 | CC | N | 13 | 61 | SL+R | 1 | 1 |  | LC |  | |  | 2.6 | 2.7 | 2.7 | 3.9 | 3.1 | 3.1 | 105 | 140 | 140 | 71 | 19 | 69 | 2.1 | 3.8 | 0.9 |
| M | 62 | CT | N | 11 | 64 | SL+R | 1 | 1 |  | LC |  | |  | 1.8 | 1.3 | 1.2 | 4.0 | 2.8 | 2.4 | 96 | 105 | 104 | 81 | 35 | 33 | 1.1 | 1.2 | 1.1 |
| K | 62 | TT | N | 16 | 68 | OPD | 1 | 1 |  | CRYO | after treatment recurrence of severe vasculitis kidney failure, and monoclonal gammopathy | |  | 2.6 | 2.9 | 1.8 | 6.0 | 9.4 | 4.9 | 216 | 218 | 181 | 38 | 26 | 14 | 2.2 | 2.1 | 2.0 |
| K | 47 | CT | N | 11 | 64 | SL+R | 1 | 1 |  | LC |  | |  | 1.5 | 1.5 | 2.2 | 3.8 | 5.6 | 5.6 | 168 | 196 | 184 | 71 | 19 | 23 | 2.3 | 3.2 | 2.7 |
| K | 47 | CT | N | 5 | 72 | OPD+R | 1 | 1 |  | LC |  | |  | 1.5 | 1.3 | 1.3 | 2.0 | 2.7 | 3.7 | 45 | 46 | 44 | 78 | 25 | 17 | 2.6 | 3.8 | 2.5 |
| M | 46 | TT | N | 10 | 66 | OPD+R | 1 | 1 |  | LC, CRYO, HCC |  | |  | 2.5 | 1.7 | 1.6 | 2.5 | 2.2 | 1.1 | 60 | 57 | 52 | 72 | 22 | 22 | 1.3 | 2.1 | 2.7 |
| M | 55 | CT | TN | 0,5 | 68 | OPD+R | 1 | 1 |  | LC, CRYO | lymphoma after treatment | |  | 0.6 | 0.4 | 0.9 | 2.8 | 2.6 | 3.1 | 82 | 96 | 136 | 74 | 6 | 8 | 1.0 | 1.3 | 0.7 |
| M | 55 | CC | R | 7 | 61 | SL+R | 1 | 1 |  | LC |  | |  | 2.1 | 1.6 | 1.9 | 2.5 | 2.3 | 3.2 | 103 | 148 | 86 | 152 | 51 | 49 | 1.2 | 1.4 | 1.7 |

Footnote for Table S2

^a^, *IFNL* rs12979860 genotype; ^b^, clinical history: TN; treatment naive; R, relapse after IFN treatment; N, IFN treatment ineffective; I, interrupted IFN treatment; ^c,^ DAA regiment: SL, sofosbuvir, ledipasvir; OPD, ombitasvir, paritaprevir, ritonavir, dasabuvir; S, sofosbuvir; R, ribavirin; ^d^, HCV-RNA in peripheral mononuclear cells (PBMC) at the follow up, results for total HCV-RNA and replicative HCV-RNA (-) strand; ^e^, clinical features before DAA therapy: LC, liver cirrhosis; CRYO, cryoglobulinemia; HCC, hepatocellular carcinoma; ^f^, adverse events recorded during therapy and at the follow up, for the two patients with HCC at the start of treatment no cancer recurrence was observed during follow up. Lymphocyte, neutrocyte, platelet counts, alanine aminotransferase (ALT) levels and neutrocyte to lymphocyte ratios (NLR) are given for baseline (0), after 12 weeks at the end of treatment (EOT), and at the follow up (F); yrs, years; wks, weeks.

**Fig. S2** Changes in ALT levels in groups of patients with or without HCV-RNA (-) strand

Alanine transaminase (ALT) was monitored during 12 weeks of DAA treatment and up to 60-72 weeks (Follow up) after start of therapy. Points show median values, whiskers represent percentiles (25^th^ to 75^th^). Filled circles, patients with HCV-RNA (-) strand in PBMCs; empty squares, patients without HCV-RNA (-) strand detected in PBMCs.

No significant differences between groups of patients for all time points were found, as calculated with two sided Mann-Whitney U test.

**Fig. S3** Changes in lymphocyte counts in groups of patients with or without adverse events

Patients were monitored during 12 weeks of DAA treatment and up to 60-72 weeks (Follow up) after start of therapy. Adverse events recorded during this time are given in Table S1. Points show median values, whiskers represent percentiles (25^th^ to 75^th^). Filled circles, patients with adverse events reported; empty squares, patients without adverse complications. *, significant (P<0.05) differences between groups of patients at a given time point calculated with two sided Mann-Whitney U test.

**Fig. S4** Changes in neutrocyte counts in groups of patients with different IFNL genotypes

Neutrocyte counts were recorded during 12 weeks of DAA treatment and up to 60-72 weeks (Follow up) after start of therapy. Points show median values, whiskers represent percentiles (25^th^ to 75^th^). Filled circles, patients with rs12979860 CC; empty squares, patients with rs12979860 CT and TT genotypes. *, significant (P<0.05) differences between groups of patients at a given time point calculated with two sided Mann-Whitney U test.

**Fig. S5** Time-course changes in neutrocyte and lymphocyte counts and values of inflammation markers for all patients

Neutrocyte (**a**) and lymphocyte (**b**) counts as well as NLR (**c**), SII (**d**), PLR (**e**) and MPV (**f**) are shown for all patients enrolled in the study. Points show median values, whiskers represent percentiles (25^th^ to 75^th^). Patients were followed for 60-72 weeks (Follow up) after start of DAA therapy. NLR, neutrocyte to lymphocyte ratio; SII, systemic immune-inflammation index; PLR, platelet to lymphocyte ratio; MPV, mean platelet volume; *, significant (P<0.05) differences between different time points calculated with two sided sign test for matched pairs.

**Fig S6** Changes in SII, PLR and MPV values in groups of patients with or without HCV-RNA (-) strand

SII (**a**), PLR (**b**) and MPV (**c**) values were recorded during 12 weeks of DAA treatment and up to 60-72 weeks (Follow up) after start of therapy. Points show median values, whiskers represent percentiles (25^th^ to 75^th^). Filled circles, patients with HCV-RNA (-) strand in PBMCs; empty squares, patients without HCV-RNA (-) strand detected in PBMCs. SII, systemic immune-inflammation index; PLR, platelet to lymphocyte ratio; MPV, mean platelet volume *, significant (P<0.05) differences between groups of patients at a given time point calculated with two sided Mann-Whitney U test.

**Table S3** Comparison of groups of patients with or without total HCV-RNA in PBMCs

| Characteristics | | | HCV-RNA in PBMC | | P value^a^ | OR (CI 95%) | P value^b^ |
| --- | --- | --- | --- | --- | --- | --- | --- |
|  |  |  | Present (n=31) | Absent (n=11) |  |  |  |
| Age | | | 55 (29-66) | 59 (39-70) | 0.136 |  |  |
| Sex (Female) | | | 16 (52%) | 5 (45%) | 1.000 |  |  |
| Liver cirrhosis ^c^ | | | 22 (71%) | 8 (73%) | 1.000 |  |  |
| HCC ^c^ | | | 3 (10%) | 0 | 0.554 |  |  |
| Cryoglobulinemia ^c^ | | | 7 (23%) | 2 (18%) | 1.000 |  |  |
| Treatment naiive | | | 7 (23%) | 3 (27%) | 1.000 |  |  |
| Duration of CHC [years] ^d^ | | | 11 (0.5-18) | 10 (2-19) | 0.735 |  |  |
| HCV-RNA [kIU/mL] ^c^ | | | 817 (3.1-17650) | 1027.5 (16.3-37900) | 0.865 |  |  |
| DAA treatment | OBV/PTV/r+DSV±RBV | | 14 (45%) | 9 (82%) | 0.075 |  |  |
|  | SOF/LDV+RBV | | 15 (48%) | 2 (18%) | 0.151 |  |  |
|  | SOF+RBV | | 2 (5%) | 0 | 1.000 |  |  |
| IFNλ3 rs12979860 genotype | | CC | 4 (13%) | 4 (36%) | 0.174 |  |  |
|  |  | TT | 8 (26%) | 1 (10%) | 0.403 |  |  |
|  |  | CT | 19 (61%) | 6 (55%) | 0.732 |  |  |
| Adverse events ^e^ | |  | 8 (26%) | 2 (18%) | 1.000 |  |  |
| Normalization of ALT at follow up | | | 24 (77%) | 10 (91%) | 0.656 |  |  |
| Δ neutrocyte count (EOT –baseline) [x10^9^ cells/L] | | | 0.16  (-1.22-4.64) | 0.69  (-0.57-3.32) | **0.035** |  |  |
| Δ lymphocyte count (EOT –baseline) [x10^9^ cells/L] | | | -0.19  (-1.42-1.05) | -0.33  (-0.81-0.10) | 0.154 |  |  |
| 0wk NLR | | | 1.6 (0.5 -7.6) | 1.5 (0.8-5.4) | 0.932 | 1.12 (0.63-2.00) | 0.694 |
| 4wk NLR | | | 1.6 (0.6 -5.4) | 2.2 (1.0-59.0) | 0.098 | 0.88 (0.56-1.38) | 0.579 |
| 8wk NLR | | | 1.6 (0.8-5.2) | 2.0 (1.4-8.4) | 0.091 | 0.83 (0.51-1.34) | 0.426 |
| 12wk NLR (EOT) | | | 1.9 (0.6-6.2) | 2.5 (1.3-9.3) | 0.138 | 0.79 (0.51-1.2) | 0.260 |
| 24wk NLR | | | 1.5 (0.6-3.9) | 1.9 (1.5-4.6) | **0.043** | 0.56 (0.23-1.38) | 0.191 |
| 48wk NLR (SVR24) | | | 1.6 (0.5-5.7) | 1.9 (1.5-4.6) | 0.131 | 0.83 (0.44-1.57) | 0.560 |
| 60-72wk (Follow up) | | | 1.5 (0.7-5.6) | 1.7 (0.8-4.5) | 0.631 | 1.05 (0.53-2.05) | 0.894 |

ALT, alanine aminotrasferase; CHC, chronic hepatitis C; EOT, end of treatment; NLR, neutrocyte to lymphocyte ratio; OBV/PTV/r+DSV±RBV, ombitasvir, paritaprevir, ritonavir, dasabuvir, ribavirin; SOF/LDV+RBV, sofosbuvir, ledipasvir, ribavirin; SOF+RBV, sofosbuvir, ribavirin; OR, odds ratio; PBMCs, peripheral blood mononuclear cells; CI, confidence intervals. For quantitative data median values with minimal maximal range are given. ^a^ for difference between groups with and without HCV-RNA in PBMCs, two-sided Fisher’s exact test for categorical data, two-sided Mann-Whitney U test with continuity correction for quantitative data; ^b^ logistic regression analysis adjusted for age and sex; ^c^ before DAA therapy; ^d^, from the diagnosis of CHC to the start of DAA treatment; ^e^, adverse events during therapy and follow up.

**Table S4** Comparison of MPV, PLR and SII values between groups of patients with or without HCV-RNA in PBMCs

| Characteristics | HCV-RNA in PBMC | | P value ^a^ | HCV-RNA (-) strand in PBMC | | P value ^a^ |
| --- | --- | --- | --- | --- | --- | --- |
|  | Present (n=31) | Absent (n=11) |  | Present (n=29) | Absent (n=13) |  |
| 0wk MPV | 11.2 (9.5-13.4) | 10.9 (9.4-12.5) | 0.778 | 11.1 (9.5-13.4) | 11.2 (9.4-12.5) | 0.893 |
| 4wk MPV | 10.8 (9.3-12.2) | 10.6 (8.7-11.8) | 0.241 | 10.7 (9.3-12.2) | 10.8 (8.7-11.8) | 0.568 |
| 8wk MPV | 10.5 (9.4-12.0) | 10.7 (9.1-11.5) | 0.323 | 10.5 (9.4-12.0) | 10.9 (9.8-11.9) | 0.441 |
| 12wk MPV (EOT) | 10.5 (9.3-12.2) | 10.6 (8.9-11.7) | 0.988 | 10.65 (9.3-12.2) | 10.4 (8.9-11.7) | 0.709 |
| 24wk MPV | 11.1 (9.9-12.0) | 10.9 (9.8-11.9) | 0.876 | 11.1 (9.9-12.0) | 10.9 (9.8-11.9) | 1.000 |
| 48wk MPV | 11.1 (9.5-12.6) | 10.9 (9.8-11.9) | 0.695 | 11.1 (9.5-12.6) | 10.7 (9.1-11.5) | 0.750 |
| 60-72wk MPV  (Follow up) | 11.0 (9.4-12.7) | 11.1 (8.9-12.2) | 0.978 | 10.8 (9.4-12.7) | 11.1 (8.9-12.2) | 0.591 |
| 0wk PLR | 69.4 (24.0-310.2) | 66.5 (29.0-466.1) | 0.822 | 61.8 (24.0-310.2) | 67.8 (29.0-466.1) | 0.591 |
| 4wk PLR | 75.3 (26.6-192.8) | 77.7 (32.7-1203.3) | 0.498 | 75.3 (26.6-192.8) | 77.7 (32.7-1203.3) | 0.346 |
| 8wk PLR | 86.0 (24.5-244.4) | 95.7 (39.6-461.5) | 0.322 | 84.9 (24.5-164.0) | 98.7 (39.6-461.5) | 0.056 |
| 12wk PLR (EOT) | 88.6 (33.3-228.6) | 120.8 (40.7-684.7) | 0.124 | 88.6 (33.3-228.6) | 120.8 (40.7-684.7) | 0.082 |
| 24wk PLR | 74. (20.9-193.4) | 73.5 (33.3-737.0) | 0.778 | 70.6 (20.9-193.4) | 91.1 (33.3-737.0) | 0.372 |
| 48wk PLR | 69.4 (24.0-194.0) | 73.5 (33.0-717.0) | 0.429 | 67.3 (14.8-193.0) | 91.2 (33.0-717.0) | 0.159 |
| 60-72wk PLR  (Follow up) | 72. (32.5-205.9) | 77.1 (25.8-487.1) | 0.714 | 66.4 (32.5-205.9) | 78.2 (25.8-487.1) | 0.318 |
| 0wk SII | 138 (64-1570) | 186 (38-2537) | 0.652 | 141 (64-1570) | 179 (38-2537) | 0.610 |
| 4wk SII | 464 (82-21311) | 226 (43-915) | 0.138 | 501 (82-21311) | 214 (43-698) | **0.026** |
| 8wk SII | 233 (104-5736) | 193 (41-1038) | 0.365 | 291 (104-5736) | 186 (41-833) | 0.077 |
| 12wk SII (EOT) | 383 (109-4580) | 220 (70-1405) | 0.233 | 406 (109-4580) | 219 (70-1405) | 0.060 |
| 24wk SII | 223 (69- 4352) | 199 (20-653) | 0.204 | 295 (69-4352) | 169 (20-649) | **0.048** |
| 48wk SII | 222 (68-3999) | 206 (20-851) | 0.233 | 296 (68-3999) | 185 (19-851) | 0.068 |
| 60-72wk SII  (Follow up) | 213 (71-1559) | 212 (34-860) | 1.000 | 233 (71-1559) | 205 (34-850) | 0.468 |

EOT, end of treatment; MPV, mean platelet volume, [fL]; PBMCs, peripheral blood mononuclear cells; PLR, platelet to lymphocyte ratio; SII, systemic immune-inflammation index; median values with minimal maximal range are given. ^a^ two-sided Mann-Whitney U test with continuity correction for comparison between patients with and without total HCV-RNA or HCV-RNA (-) strand; statistically significant p values (P<0.05) are shown in bold.
